# Is Anxiety related to oral examination scores of Anatomy and Physiology? A study of pre-clinical MBBS and BDS Students

**DOI:** 10.12669/pjms.40.9.8544

**Published:** 2024-10

**Authors:** Ambreen Surti, Ambreen Usmani, Muhammad Raza Sarfraz, Shazia Shakoor

**Affiliations:** 1 Ambreen Surti, MBBS, MPhil, MHPE Assistant Professor, Department of Biological and Biomedical Sciences, Aga Khan University, Karachi, Pakistan; 2 Prof. Dr. Ambreen Usmani, MBBS, MPhil, PGD-E, MCPS-HPE, PhD, FCPS- Anatomy Professor Anatomy & Principal Jinnah Medical and Dental College, Karachi, Pakistan; 3 Muhammad Raza Sarfraz, MBBS Alumni Bahria University Health Science & Visiting student, Oxford University Medical School, Bahria University Health Science (BUHSCK) Karachi, Karachi, Pakistan; 4 Prof. Dr. Shazia Shakoor, MBBS, MPhil, PhD Head of Department Physiology Bahria University Health Science (BUHSCK) Karachi, Karachi, Pakistan

**Keywords:** Medical Students, Test Anxiety, Oral examination, Undergraduate, University, Cross-sectional study

## Abstract

**Objective:**

To assess anxiety levels among 1^st^ and 2^nd^ year MBBS and 1^st^ year BDS students in oral examinations of anatomy and physiology and to compare the anxiety scores among students of 1^st^ and 2^nd^ year MBBS and 1^st^ year BDS.

**Methods:**

This cross-sectional study was conducted on 231 undergraduate (MBBS and BDS) students of University from Karachi durning December 2022 to May 2023. Westside anxiety scale was used to assess anxiety levels in students and the scores were compared with oral examination scores using One-way analysis of variance.

**Results:**

Extremely high levels of anxiety was noted in relation to oral examination of Anatomy with a mean score of 25±3.801 while a mean of 30.00±5.170 was noted in Physiology in BDS students. However, significant results were observed on comparing Anatomy and Physiology oral examination scores among 1^st^ and 2^nd^ year students.

**Conclusion:**

In summary higher levels of test anxiety, especially for oral examinations was observed in MBBS students as compared to BDS students. It was also observed that anxiety levels were much higher for Anatomy oral examinations as compared to those for Physiology.

## INTRODUCTION

Medical education is known to be emotionally and physically demanding. Exposing the students to excessive workload and psychosocial stressors, perceived to be norms of a medical school, are in reality a major source of anxiety, depression and burnout leading to substandard patient care and safety.^1^,^2^

Literature defines test anxiety as *“an anxiety disorder which is characterized by cognitive, behavioral, and affective responses of fear to a performance/test situation, which may lead to a worsened performance or even failure of the examination”*.^3^ Globally, it impacts 284 million people, with a female preponderance. Systematic review by Ahmed et.al., noted anxiety to be one of the most common mental health problems among university students with one-third suffering from anxiety disorder resulting in poor academics.^4^

Prevalence of anxiety among medical students worldwide is 33.8%. In Pakistani medical students, frequency of anxiety (88.4%) surpasses that of depression (75%).^4^,^5^ Another study noted a 31.9% prevalence of among first year medical students.^6^

A cross-sectional study on medical and dental students observed higher test anxiety in female students at private sector universities.^7^ Another study on medical students observed significant rise in salivary cortisol levels in oral examination as compared to written examination.^8^

Oral examinations, one of the common assessment methods in public and private sector universities in Pakistan, invigorates anxiety among students. Anxiety in a medical student can seriously hamper their communication skills and significantly impact physician-patient relationships. Anxiety, stress, and depression among medical graduates have been measured and documented, however, literature is limited where anxiety scores are compared with oral examination scores. This paper will add to the body of literature by measuring the association between these two strongly interdependent variables and simultaneously influence policy makers to come up with assessment strategies which can reduce stress and anxiety among students. This study aims to assess anxiety levels among 1^st^ and 2^nd^ year MBBS and 1^st^ year BDS students in oral examinations and to compare the anxiety scores among students.

## METHODS

This cross-sectional study was conducted on 231 undergraduate (MBBS and BDS) students of Bahria University Health Sciences Campus, Karachi (BUHSCK) from December 2022 to May 2023.

### Sampling Technique:

Purposive.

### Ethical approval:

Approval was obtained from Ethical Review Committee of BUHSCK (ERC 21/2022, Dated: August 21, 2022).

### Study subjects:

Written and understood consent was taken from the participants. Data was coded for confidentiality. From the total sample, 81 students were from second year MBBS, 118 and 32 from 1^st^ year MBBS and BDS respectively.

### Inclusion Criteria:

All study participants who were in good general health and had appeared in modular examinations.

### Exclusion Criteria:

- Students on psychiatric treatment
- Students with a history of drug abuse
- Students with any other chronic illness

### Tool:

Westside anxiety scale was used to assess anxiety levels in students. The students were explained the definition of anxiety as *“a feeling of worry, fear, uneasiness and dread.”* Using the scale anxiety levels were assessed as shown in Table-I.

**Table-I:** Relation of anxiety level with scores achieved on the Westside anxiety scale

Anxiety Level	
Comfortably low-test anxiety	0 – 1.9
Normal or average test anxiety	2.0 -2.5
High normal test anxiety	2.5 -2.9
Moderately high-test anxiety	3.0 -3.4
High test anxiety (half or more items rated 4 =high)	3.5-3.9
Extremely High test anxiety (items rated 4=high and 5= extreme)	4.0 -5.0

Following modular curriculum, data was collected at the end of 12-week module 2 of 1^st^ year MBBS and BDS comprising of musculoskeletal and head & neck respectively. While data from 2^nd^ year was collected at the end of module 5 (head and neck and neuroanatomy). Oral examination by a panel of four examiners and is a one-to-one interaction with the student for 4-5 minutes. The scale was filled 1 day after the modular oral examinations by all participants.

### Statistical analysis:

Data was analyzed by SPSS version 25. All quantitative variables were expressed as Mean±SD while one way analysis of variance (ANOVA) was used to compare anxiety levels among 1^st^ and 2^nd^ year MBBS and 1^st^ year BDS students with their oral examination scores and chi square was used to observe the association between anxiety levels and examination results.

## RESULTS

The mean age of participants was 20.06 ± 1.13. Most of the participants were female (n=148) while 83 were male.

It was observed that seventeen students (41.5%) in 2^nd^ year MBBS and twelve students (29.3%) each in 1^st^ year MBBS and BDS reported extremely high anxiety. In contrast, only one student (2.5%) from the 1^st^ year BDS, twenty-seven students (67.5%) from the 1^st^ year MBBS, and twelve students (30%) from the 2^nd^ year MBBS reported comfortably low-test anxiety. These findings indicated a significant result (p-value= 0.003), showing that anxiety levels, in comparison, are extremely high among 2^nd^ year students.

Among the total number of participants, only 31 were unable to pass their exams. Out of this maximum number of failures were from 2^nd^ year MBBS (87%) while only two students each were from 1^st^ year of MBBS and BDS. Of the students who failed, 16 suffered extremely high anxiety and 12 had high-test anxiety. Among the 200 students who passed, 32 reported high normal test anxiety, 36 normal or average test anxiety, and 40 reported comfortably low-test anxiety. This difference was highly significant (p-value 0.000).

### Comparison of oral Anatomy and Physiology examination scores of BDS students:

**Fig.1:**
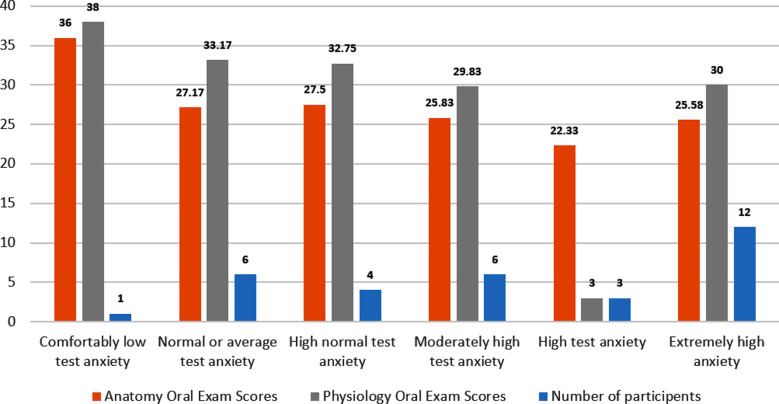
Comparison of oral Anatomy and Physiology examination scores of BDS students.

Out of 32 ***students*** 12 students felt extremely high anxiety during oral examinations. A mean score of 25±3.801 was observed in Anatomy while a mean of 30.00±5.170 was observed in Physiology oral examination with non-significant results (p-value = 0.173). Maximum marks, 36/40, were obtained by students with comfortably low-test anxiety levels in anatomy as compared to 38/40 marks in physiology as shown in Fig.1. This difference in marks in relation to anxiety levels indicate, that students feel more anxious during Anatomy examinations.

### Comparison of oral Anatomy and Physiology examination scores of 1^st^ and 2^nd^ year MBBS students:

Significant results, where a maximum score of 33 with a Mean±SD of 26.42±5.368 was noted in Anatomy oral examinations in extremely high-test anxiety, as compared to physiology (Table-II). Highly significant difference was observed between oral examination scores of Anatomy and Physiology as shown in Table-III.

**Table-II:** Comparison of oral Anatomy and Physiology examination scores of 1^st^ year MBBS students.

		Anatomy Oral Exam Scores (40 marks)			Physiology Oral Exam Scores (40 marks)			p-value

	n (118)	Min	Max	Mean±SD	Min	Max	Mean ±SD	
1.0-1.9 Comfortably low-test anxiety	27	20	38	29.81± 4.844	25	40	33.33±4.297	0.004
2.0-2.5 Normal or average test anxiety	23	12	37	28.74± 5.707	25	38	32.30±3.686	
2.5-2.9 High normal test anxiety	16	12	35	26.81± 6.635	25	37	32.25±3.357	
3.0-3.4 Moderately high (some items rated 4=high	25	16	33	24.60 ±5.017	22	40	31.56±4.709	
3.5-3.9 High test anxiety (half or more of the items rated 4=high)	15	15	30	24.33 ±4.716	25	38	31.47±3.758	
4.0-5.0 Extremely high anxiety (items rated 4=high and 5=extreme)	12	16	33	26.42 ±5.368	25	40	33.33±4.579	

**Table-III:** Comparison of oral anatomy and physiology examination scores of 2^nd^ year MBBS students.

	Anatomy Oral Exam Scores (40 marks)				Physiology Oral Exam Scores (40 marks)			p-value

	n (81)	Min	Max	Mean ±SD	Min	Max	Mean ±SD	
1.0-1.9 Comfortably low test anxiety	12	20	38	30.92±4.899	26	39	32.67±4.355	0.000
2.0-2.5 Normal or average test anxiety	7	26	38	30.71±4.112	22	39	32.71±5.880	
2.5-2.9 High normal test anxiety	12	13	33	27.83±6.073	30	39	34.50±3.503	
3.0-3.4 Moderately high (some items rated 4=high	14	20	34	25.64±5.048	25	37	30.79±4.003	
3.5-3.9 High test anxiety (half or more of the items rated 4=high)	19	11	37	23.89±6.100	25	37	30.53±4.647	
4.0-5.0 Extremely high anxiety (items rated 4=high and 5=extreme)	17	16	33	22.88±5.023	21	37	29.12±4.196	

## DISCUSSION

Assessment, being the main method of grading the students, is a major concern for deteriorating mental health in students due to the failure of achieving the required competency levels despite putting in the best efforts.^9^ The majority of the participants in our study were in their 1^st^ year of MBBS with an average age of 20.06 years. Overall, 2^nd^ year MBBS students reported higher levels of anxiety, and most of them were unable to pass their exams. Twelve students from each, 1^st^ years BDS and MBBS, as well as 17 from 2^nd^ year MBBS, reported extremely high exam anxiety. Psychologists refer to students’ anxiety as *Examination Phobia and Fear of Exam*. Examination anxiety is a type of worry that causes pupils to avoid fearful circumstances such as exams.^10^ Minimal level of this anxiety is productive, however, when exceeded, it hampers the students’ performance during examinations.^10^,^11^ This fact is also evident from the current study results where students suffering from high anxiety levels were unable to clear their examination. This can result from various causes such as peer pressure, pressure of passing exams, lack of preparation, family conditions, female gender, year of study, over expectations and extensive course load.^11^,^12^ Examination anxiety being more severe and long-lasting, can seriously hinder a student’s performance.^12^ The most likely cause of extremely high levels of anxiety in the current study especially in 2^nd^ year MBBS students, pertains to the fact that module 5 is most lengthy and requires clear understanding and conceptualization of neuroanatomy. Moreover, Anatomy includes gross anatomy, histology, and developmental anatomy as well. Thus, incurring higher levels of anxiety and apprehension among students. Ironically examination-related anxiety and stress, to date remain untreated and have never been addressed seriously.^1^ Both examination fear and anxiety have a negative association with academic performance as well as the mental and physical well-being of the students.^2^,^3^,^11^,^12^ Since, viva examinations are an important aspect of the medical school system, students’ fear of viva or oral examination inevitably causes exam anxiety.^11^

As observed in literature, our results showed that 12 out of 32 students exhibited extreme anxiety during viva examination.^2^,^4^,^9^ An Iraqi study reported that more than half the students experienced symptoms of anxiety like severe sweating and stomach ache, before a major examination.^13^ Our study results also showed that BDS students suffered from higher levels of anxiety when faced with Anatomy oral examination as compared to Physiology. A study conducted by Saheen et al., on 104 medical students, with a mean age of 21.26 ± 1.23, found significant correlation (p-value < 0.001) between anxiety levels and academic performance.^13^ The data of the present study showed the prevalence of extremely high anxiety (41.5%) in 2^nd^ year MBBS students as compared to 1^st^ year MBBS and BDS students. The results are in accordance with a study conducted by Hussain et al.^9^, on students of medicine, dentistry, and pharmacy, where maximum number of anxiety cases were noted in 2^nd^ year MBBS students. This is likely because medical students encounter significant stress as they are more conscious about their academic performance in pre-clinical subjects prior to clinical exposure.^9^ A study using Westside Anxiety Scale by Bonna et al. on medical students, reported that most participants (30%), had normal or medium test anxiety. This variation might be due to their different methodology and the time of their study, which was done during the COVID-19 pandemic.^10^

A German study conducted on 625 medical students, observed initial levels of anxiety on joining the session was 6% which later increased to 10%, indicating that in the initial days, test and examinations were a farfetched idea, therefore, low anxiety levels whereas the levels escalated as the examination days grew closer.^14^ Current study results also corroborates with a Spanish study which observed a decline in academic performance of students in cases of higher test anxiety.^15^ Contrary results are reflected in a study conducted by Grochowski et al on 60 medical students taking 13-weeks Gross Anatomy course. Using Beck’s Anxiety inventory, the authors reported minimal anxiety levels which contradicted the verbal version of students on the exit interviews.^16^

In current study, students expressing high anxiety were unable to clear the examination. While contradictory results were noted by a study in Dhaka where mild anxiety was observed in students who failed to clear their exams as compared to those who experienced severe anxiety while taking supplementary examinations.^10^ This discrepancy may be due to the inclusion of graduate and postgraduate medical students. Studies indicate that the incidence of anxiety among medical students varies greatly depending on the group investigated and the instruments utilized. In contrast to our research, another study using Hamilton anxiety rating scale, observed 25% mild and 4% had moderate to severe anxiety among medical studnets.^17^ A recent review paper noted anxiety levels ranging from 7.7-65.5% among 2^nd^ year female medical students (52.58%).^2^

Comparison of anxiety levels of students in Anatomy and Physiology oral examinations in the current study showed significant results. These are corroborated by an Iraqi study which highlighted heavy workload and lack of sleep as the root cause of anxiety, thereby stressing on introduction of anxiety reduction programs in medical schools.^9^ Another German study on 196 first-year medical students, studied the effect of hypnosis in reducing anxiety. The study reported that students experienced higher level of anxiety as the test approached closer and therefore one hour of hypnosis in such cases reduced the stress to considerable level.^3^ A study conducted on 70 pre-clinical students compared the levels of anxiety before a written and oral examination and noted that stress and anxiety levels among students increased steadily especially in oral examinations as the day to the examinations got closer.^18^

The exact cause of this anxiety is still unclear, but many researchers have mentioned extensive course load as an obvious reason.^19^ Our results are also consistent with a prevalence study conducted at a Pakistani University, which concluded that stress levels were significantly higher in first- and second-year medical students as compared to those in third and fourth year.^20^

Oral examinations play a significant part in pre-clinical years to develop communication skills, but many students feel stress and experience various levels of examination related anxiety.^11^ Our findings of anxiety during oral examination are consistent with many national and international studies. Many students of the current study have language barriers, which adds to the overall level of anxiety. Literature suggests course workload and fear of disappointing the family as the underlying causes, hence the need to devise effective tools for mentoring and counselling is required.^13^ Literature also suggests supportive measures, an increase in the number of formative assessments and a detailed briefing about how the examination will be conducted, may act as a major stress and anxiety reliever for the students.^21^

To the best of our knowledge this is one of the few studies which has compared the levels of anxiety among MBBS and BDS students with oral examinations scores. Moreover, it opens horizons for further research to relate factors responsible for differences in anxiety levels between the two disciplines and development of assessment methodologies which can incur better academic results without hampering the mental health.

### Limitation of the study:

Results cannot be generalized as it’s a single center study. Public and private sector students may have different precursors of anxiety resulting in a different impact on academic performance.

## CONCLUSION

In conclusion, higher levels of anxiety were found among MBBS students as compared to BDS students. It was also observed that anxiety levels were increased several folds in case of oral Anatomy examination. Further studies with larger cohorts and in different settings should be conducted to generalize results. Current study results can help academicians to think about less stressful assessment methods or to train faculty in such a way that oral examinations become more structured and be conducted in a conducive, less stressful environment.

### Authors Contribution:

**AS:** Conceived, designed, prepared manuscript, data analysis and responsible for the integrity and accuracy of the manuscript.

**AU:** Review, analysis and final approval of manuscript.

**RS:** Data collection, data analysis and review.

**SS:** Literature search, data collection and analysis.
